# A comparative evaluation of parafoveal and perifoveal macular vessel density loss in glaucoma using 3 × 3 mm OCTA scans

**DOI:** 10.1038/s41598-026-36230-w

**Published:** 2026-01-22

**Authors:** David Garcia Kahmeyer, Christian Mardin, Robert Lämmer, Bettina Hohberger, Julia Schottenhamml

**Affiliations:** Department of Ophthalmology, Universitätsklinikum Erlangen, Friedrich-Alexander-Universität Erlangen-Nürnberg, Erlangen, Germany

**Keywords:** Diseases, Medical research

## Abstract

In a prior study we demonstrated the strong performance of convolutional neural networks (CNNs) in distinguishing healthy from glaucomatous eyes and subsequent analysis suggested that CNNs rely on the vessel density (VD) of the perifoveal area for making their predictions. Those findings are of particular interest because current 3 × 3 mm macular optical coherence tomography angiography (OCTA) analysis mostly focuses on the parafoveal area, excluding the perifoveal region. The aim of this study is to further investigate this observation and evaluate whether perifoveal VD offers greater diagnostic accuracy in differentiating healthy from glaucomatous eyes than parafoveal VD. For conducting this study, 3 × 3 mm macular OCTA scans where acquired by the Spectralis OCT II device of 352 eyes, consisting of 198 glaucomatous and 154 healthy eyes. The different layers of the retina were automatically segmented by the manufacturer’s software and sectioned into the superficial vascular plexus (SVP), intermediate capillary plexus (ICP) and deep capillary plexus (DCP). Support vector machines (SVMs) were then trained on perifoveal and parafoveal VD for each plexus, whereby parafoveal VD was computed on 12 sectors within a 1.45 mm radius centered at the fovea, while parafoveal VD was trained on 4 quadrants covering the remaining area of the scan. 10-fold cross-validation was used to select the SVMs with the highest mean area under the receiver operating characteristic curve (AUROC) and evaluated on a held-out test set. Differences in AUROC between methods were pairwise statistically tested with Bonferroni correction. It was found that perifoveal VD outperformed parafoveal VD across all plexuses, with the highest improvement observed in the SVP (AUROC perifoveal 0.943 vs. parafoveal 0.897, *p* = 0.025), followed by the ICP (AUROC perifoveal 0.872 vs. parafoveal 0.851) and DCP (AUROC perifoveal 0.795 vs. parafoveal 0.786). Our results demonstrate that perifoveal VD in 3 × 3 mm macular OCTA scans offers a higher diagnostic performance in distinguishing healthy from glaucomatous eyes than parafoveal VD. These findings suggest that incorporating the perifoveal region into macular OCTA analysis could yield additional information on glaucoma, especially when focusing on the SVP. Furthermore, our findings propose to reconsider the value of 3 × 3 mm macular scans in VD analysis, whose utility appears to be underestimated.

## Introduction

 Glaucoma is a chronic, progressive optic neuropathy and one of the leading causes of irreversible vision loss worldwide^1^. As of 2020, around 76 million individuals were affected by the disease globally, with projections indicating an increase to 111 million by 2040^2^.

The pathophysiology of glaucoma is seen as complex and not yet fully understood. Research suggests that there are multiple components responsible for the onset and progression of the disease, with a central focus on the degeneration of retinal ganglion cells (RGCs). RGCs are neurons of the central nervous system whose axons project through the retinal nerve fiber layer (RNFL) to the optic nerve. They exit the eye at the lamina cribrosa, a relatively weak point within the sclera. The mechanical theory suggests that elevated intraocular pressure (IOP), a major risk factor of glaucoma, can damage RGCs in this particular area and disrupt axonal transport, causing RNFL thinning, structural changes of the optic nerve head (ONH) and eventually visual field loss^3^. However, not every patient with high IOP develops glaucoma, whereas in some instances the disease emerges even in subjects with normal IOP. The vascular theory offers a complementary approach and suggests that impaired blood flow of the retinal and ONH vasculature, often as a result of vasospasm or autoregulatory dysfunction of blood vessels, plays a critical role in RGC loss as well and therefore contributes to the pathogenesis of the disorder^4^.

Glaucoma remains asymptomatic in its initial stages for most patients. Typical symptoms like irreversible vision loss start to present themselves when around 30–50% of RGCs have already degenerated^3,5^. This highlights the importance of early glaucoma diagnosis, which is crucial for detecting the disease early enough to slow down progression and therefore prevent vision loss from occurring^3^.

The macula is of particular interest for glaucoma diagnosis since more than 30% of RGCs are located in this area^6^. It consists of the fovea, an avascular zone in the center of the macula, the parafovea, an annulus of 2.5 mm in diameter surrounding the fovea and the perifovea, the outermost part of the macula^7^. While traditional glaucoma diagnostics focused primarily on the optic nerve and peripapillary region, present-day methods put additional emphasis on the macula, with more recent studies demonstrating its susceptibility for early glaucomatous changes in particular^8^.

Current glaucoma diagnosis therefore relies primarily on clinical examination of the ONH and macula, complemented by key tests such as tonometry, perimetry and optical coherence tomography (OCT)^1^. OCT is a non-invasive tool and has become the standard imaging modality for assessing structural damage of the ONH and retina^9^. Optical coherence tomography angiography (OCTA) was later on introduced to extend the use case of OCT by visualizing the retinal vascular system, particularly the superficial vascular plexus (SVP), intermediate capillary plexus (ICP) and deep capillary plexus (DCP)^10^.

In OCTA diagnostics, vessel density (VD) serves as a biomarker, illustrating the percentage of an area covered by blood vessels. A higher VD indicates more blood vessels being present in a particular area and can therefore refer to richer perfusion. In glaucoma, VD is usually reduced in the optic disc and macula, correlating with overall glaucoma severity such as RNFL thinning and visual field (VF) loss^11,12^.

A previous study by Schottenhamml et al.^13^ demonstrated the high diagnostic accuracy of convolutional neural networks (CNNs) in distinguishing healthy from glaucomatous eyes using 3 × 3 mm macular OCTA scans. The analysis of saliency maps, methods applied to neural networks to highlight which regions most influenced their decision, showed that CNNs seem to focus mostly on perifoveal regions for their decision making^13^. These findings are in stark contrast to conventional 3 × 3 mm macular OCTA VD analysis, which is mostly being performed on the parafoveal region and thereby excluding the perifoveal area^14–16^. Furthermore, previous research states that 3 × 3 mm macular OCTA scans might be disadvantageous for glaucoma detection compared to larger scans such as 6 × 6 mm, mainly based on the assumption that glaucomatous changes happen outside the smaller 3 × 3 mm scan window^16,17^.

The current study therefore aims to further investigate this observation and determine whether perifoveal VD in 3 × 3 mm macular OCTA scans offers higher diagnostic performance in differentiating healthy from glaucomatous eyes than parafoveal VD. To achieve this goal, support vector machines (SVMs) were trained on VD values of the parafoveal and perifoveal region across all retinal plexuses (SVP, ICP, DCP). A 10-fold cross-validation was performed and each SVM model was evaluated using the area under the receiver operating characteristic curve (AUROC) to assess their diagnostic performance and accuracy.

## Methods

### Data

For this study, 352 eyes of the Erlanger Glaucoma Registry (Erlangen Glaucoma Registry, ISSN 2191–5008, CS-2011; NTC00494923), were analysed. Out of total, 198 eyes were classified as glaucomatous, consisting of 104 male and 94 female subjects. The average age of the glaucoma cohort was 65.04 years with a standard deviation of 11.69 years. In comparison, 154 healthy eyes were included, consisting of 70 male and 84 female participants with an average age of 63.38 years and standard deviation of 13.10 years. Demographic details are further displayed in Table 1.

**Table 1:** Demographic data of the two groups (glaucoma, controls) including the number of eyes and their respective age; mean ± standard deviation.

	Number (male/female)	Age [years]
Controls	154 (70/84)	63.38 ± 13.10
Glaucoma patients	198 (104/94)	65.04 ± 11.69

**Fig. 1:**
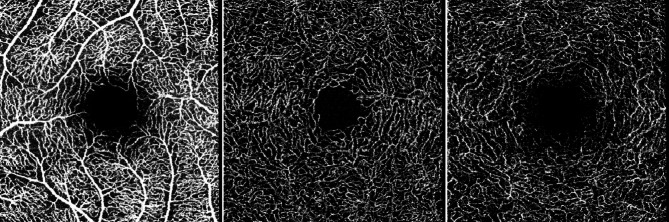
Visualization of the different plexus interfaces automatically computed and exported by the Heidelberg Eye Explorer software: Superficial vascular plexus (SVP), intermediate capillary plexus (ICP) and deep capillary plexus (DCP).

All study participants underwent thorough ophthalmological examination including automated visual field testing, slit lamp examination of the anterior and posterior eye segments, pachymetry for measuring corneal thickness, IOP assessment by Goldmann tonometry and fundus photography. Furthermore, OCT imaging of the RNFL, RGC, inner nuclear layer (INL) and Bruch’s Membrane Opening-Minimum Rim Width (BMO-MRW) was performed by Heidelberg Spectralis OCT II device (version 1.9.10.0, Heidelberg Engineering, Heidelberg, Germany, Glaucoma Premium Module). Additionally, 3 × 3 mm macular OCTA scans were acquired by the Spectralis II device, centered around the fovea at a 15 × 15° angle with a lateral resolution of 5.7 μm per pixel. The SVP, ICP and DCP were automatically computed using the Heidelberg Eye Explorer software from Heidelberg Engineering. The segmentation lines of the plexuses were verified for accuracy and manually adjusted whenever necessary (Fig. 1).

Ethical approval was obtained from the Ethics Committee of the Friedrich-Alexander-Universität Erlangen-Nürnberg (FAU) and all procedures were conducted in accordance with the Declaration of Helsinki. Prior to inclusion, written informed consent was obtained from each participant.

### Evaluation

**Fig. 2:**
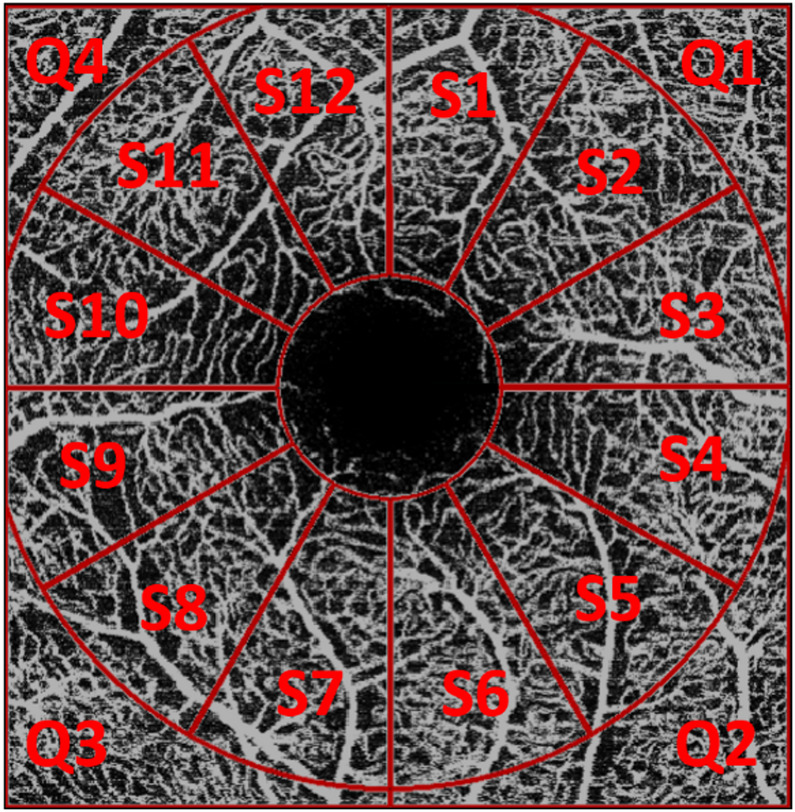
Visualization of the regions (parafoveal: 12 sectors S1-S12; perifoveal: 4 quadrants Q1-Q4) used for the computation of the VD.

For each plexus, VD was assessed in both the parafoveal and perifoveal regions. The parafoveal region was defined as a 2.9 mm diameter annulus centered on the fovea, excluding the foveal avascular zone, and was subdivided into 12 sectors of 30° each. The perifoveal region encompassed the area outside of this annulus and was divided into four quadrants, corresponding to the four corners of the image. This layout is further illustrated in Fig. 2.

The projections of the different plexuses were automatically segmented by the Heidelberg Eye Explorer software from Heidelberg Engineering and manually corrected if necessary. These projections were then exported as images and used as input to the Erlangen-Angio-Tool (EA-Tool) in order to compute the VD of each sector and quadrant. The EA-Tool was developed in MATLAB (The MathWorks, Inc., Natick, USA, R2017b) and has demonstrated high reliability and reproducibility in a previous study^18^.

The EA-Tool uses the OCTA projections of the different plexuses exported from the Heidelberg Eye Explorer as input and computes a binary image. To achieve this, the vessel structures are highlighted in a first step using a Frangi vesselness filter. Subsequently, binarization is performed using the Otsu thresholding algorithm. From these binary vessel images, vessel density is computed in the sectors and quadrants defined above by calculating the ratio of vessel pixels to all pixels in these defined regions.

The 12 parafoveal sector VD values were used as input features to train a support vector machine (SVM) classifier. The dataset was split into 60% training, 20% validation, and 20% test sets, ensuring that all eyes from a given patient were assigned exclusively to one set to prevent data leakage. A 10-fold cross-validation was performed, during which various SVM hyperparameter configurations were trained on the training set and evaluated on the validation set. The AUROC was used as the performance metric. The configuration with the highest mean AUROC on the validation set was selected for final evaluation on the test set.

This procedure was repeated for the perifoveal region using the four quadrant VD values as input. Additionally, for both parafoveal and perifoveal regions, separate SVM models were trained for each of the three plexuses.

For parafoveal VD values, SVM architectures with a polynomial kernel showed the best performance on the mean AUROC value of the validation set, whereas SVM architectures with a sigmoid kernel performed best for perifoveal VD values. Finally, the selected parafoveal and perifoveal SVM models were evaluated on the independent test set, with AUROC again used as the performance metric.

To assess whether the observed differences in AUROC values between the two regions were statistically significant, a one-way ANOVA was conducted using SPSS version 28. Post hoc multiple comparisons were performed with a Bonferroni correction to control for Type I error.

## Results

**Table 2:** Performance metrics of the 10-fold cross-validation for the SVMs trained on the perifoveal and parafoveal VD values of the different plexuses; mean ± standard deviation.

Layer / Region	AUROC	Sensitivity	Specificity	F1-Score
SVP perifoveal	0.943 ± 0.012	0.848 ± 0.032	0.926 ± 0.040	0.888 ± 0.016
SVP parafoveal	0.897 ± 0.018	0.759 ± 0.052	0.915 ± 0.059	0.822 ± 0.027
ICP perifoveal	0.872 ± 0.036	0.759 ± 0.038	0.869 ± 0.036	0.813 ± 0.033
ICP parafoveal	0.851 ± 0.035	0.780 ± 0.068	0.815 ± 0.057	0.789 ± 0.034
DCP perifoveal	0.795 ± 0.034	0.701 ± 0.041	0.805 ± 0.075	0.740 ± 0.030
DCP parafoveal	0.786 ± 0.024	0.743 ± 0.056	0.763 ± 0.043	0.740 ± 0.027

Table 2 illustrates the mean and standard deviation of the performance metrics of the 10-fold cross-validation for the SVMs trained on the perifoveal and parafoveal VD of the different plexuses. The data collected demonstrates that perifoveal VD consistently scored higher AUROC values compared to parafoveal VD, which is the case for all three plexuses (SVP, ICP, DCP). The largest difference was observed in the SVP: Perifoveal SVP VD achieved a mean AUROC of 0.943 ± 0.012 (sensitivity 85%, specificity 93%, F1-score 89%), which was notably higher than the 0.897 ± 0.018 (sensitivity 76%, specificity 92%, F1-score 82%) obtained for parafoveal VD. Higher AUROC values were also achieved for the ICP: Perifoveal ICP VD achieved a slightly higher result with an AUROC of 0.872 ± 0.036 (sensitivity 76%, specificity 87%, F1- score 81%) compared to 0.851 ± 0.035 (sensitivity 78%, specificity 82%, F1-score 79%) for parafoveal VD. Additionally, perifoveal DCP VD also outperformed parafoveal DCP VD with 0.795 ± 0.034 (sensitivity 70%, specificity 81%, F1-score 74%) compared to 0.786 ± 0.024 (sensitivity 74%, specificity 76%, F1-score 74%) although more marginally.

**Fig. 3:**
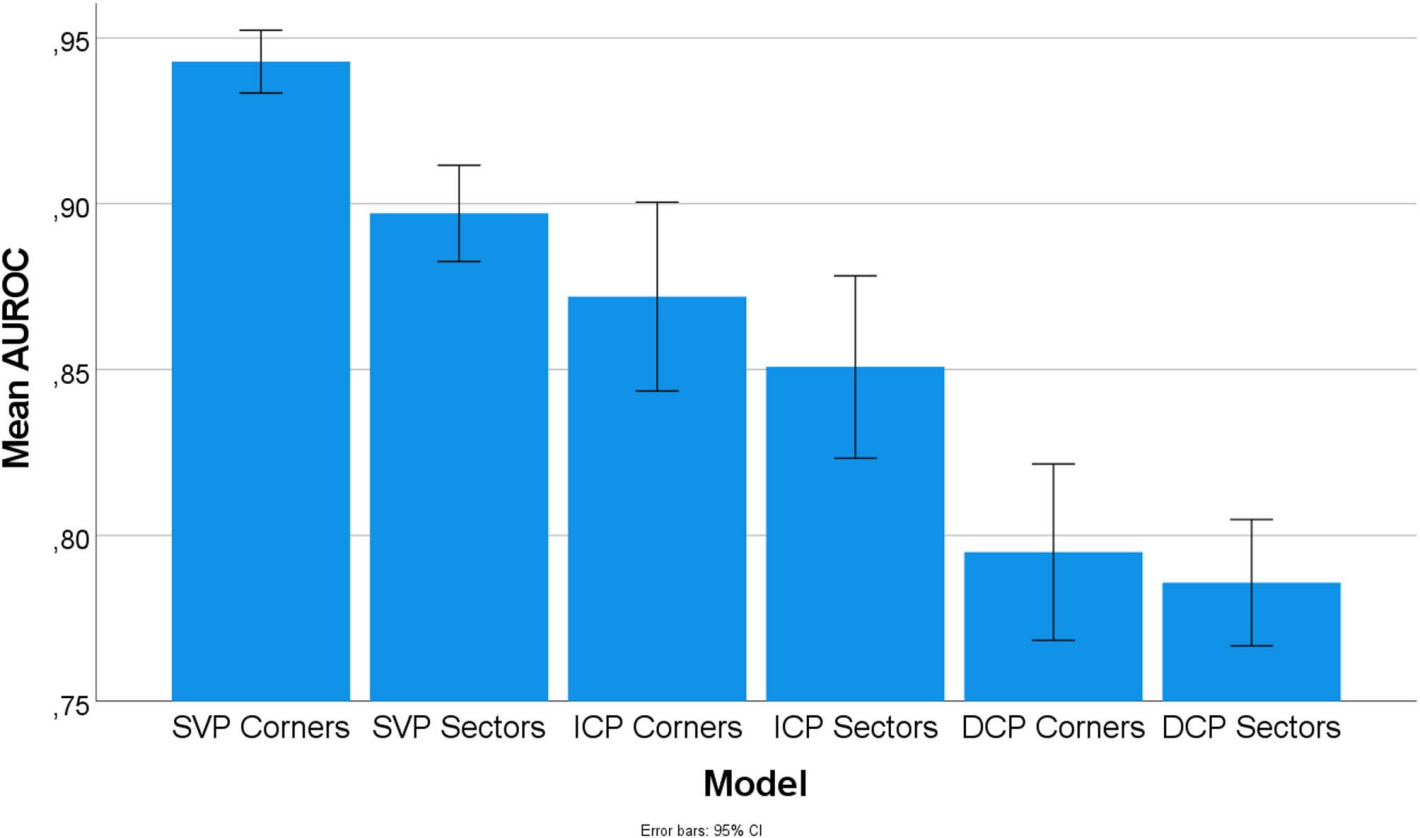
Visualization of the mean AUROC values with error bars indicating the 95% confidence interval.

These findings are further visually represented in Fig. 3, which displays the mean AUROC values with error bars indicating their 95% confidence intervals.

**Table 3:** Pairwise comparisons of the AUROC values of the 10-fold cross-validation for the different methods corrected with Bonferroni; p-values; statistically significant values are highlighted in bold.

	SVP perifoveal	SVP parafoveal	ICP perifoveal	ICP parafoveal	DCP perifoveal	DCP parafoveal
SVP perifoveal		**0.025**	**< 0.001**	**< 0.001**	**< 0.001**	**< 0.001**
SVP parafoveal			1.00	**0.022**	**< 0.001**	**< 0.001**
ICP perifoveal				1.00	**< 0.001**	**< 0.001**
ICP parafoveal					**0.003**	**< 0.001**
DCP perifoveal						1.00
DCP parafoveal						

The one-way ANOVA revealed significant differences between groups (*p* < 0.001). The p-values from the pairwise comparisons of the AUROC values, corrected using the Bonferroni method are given in Table 3. This table is used to determine if the differences in performance between the various SVMs (e.g. perifoveal SVP vs. Parafoveal SVP) are statistically significant. It indicates that perifoveal SVP significantly differed from ICP and DCP (*p* < 0.001) both in the parafoveal and perifoveal region. Furthermore, perifoveal ICP turned out significant compared to perifoveal and parafoveal DCP (*p* < 0.001). Within the parafoveal area, SVP differed from ICP (*p* = 0.022) and DCP (*p* < 0.001) respectively. Moreover, there was a significant difference in performance between the ICP and DCP.

## Discussion

This study builds upon findings of Schottenhamml et al.^13^, which highlighted the strong diagnostic performance of convolutional neural networks (CNNs) in differentiating healthy controls from glaucoma using 3 × 3 mm macular OCTA scans. A key finding of their work was the observation that CNNs focus on the border of the scans and therefore the perifoveal area for their decision making. The aim of the current study was to further investigate this observation by determining if perifoveal VD of 3 × 3 mm OCTA scans offers higher diagnostic accuracy in distinguishing healthy from glaucomatous eyes than parafoveal VD.

Our results illustrate that perifoveal VD performs significantly better than parafoveal VD in differentiating healthy and glaucomatous eyes. This could be achieved across all assessed retinal plexuses: Superficial vascular plexus (SVP), intermediate capillary plexus (ICP) and deep capillary plexus (DCP), whereby SVP results have proven to be the most significant. These findings suggest that the diagnostic performance of VD analysis is richer in the perifoveal area.

Our findings of perifoveal VD performing significantly better than parafoveal VD are in line with the broader observation that diagnostically important information can often be found in the perifoveal region. Penteado et al.^19^ compared scans of the outer macula (which tend to image the perifoveal region) with inner macular scans and found that the outer macula offers higher diagnostic accuracy than the inner macula, especially in early glaucoma. Lu et al.^20^ compared perifoveal and parafoveal VD in pre-perimetric (PPG) and early perimetric glaucoma (EG) and observed that although VD was both significantly reduced in EG, perifoveal VD was more heavily impaired in PPG than parafoveal VD. Despite both referring to 6 × 6 mm and therefore a larger scan window than the 3 × 3 mm which was used in our study, their results highlight the diagnostic relevance of the outer macula and thus the perifoveal region for early glaucoma diagnosis.

Furthermore, our observation of perifoveal SVP VD offering strong diagnostic performance is consistent with current research. Chen et al.^21^ evaluated the superficial microvasculature of the macula in glaucomatous and healthy eyes and revealed that the SVP VD was significantly decreased in individuals with glaucoma (AUROC 0.94, sensitivity 92%, specificity 80%). Takusagawa et al.^17^ examined macular perfusion across the superficial vascular complex (SVC = SVP + radial peripapillary capillary plexus), ICP and DCP and identified the largest distinction in the SVC (AUROC 0.983, sensitivity 96.7%, specificity 95%). These findings confirm our assumption that glaucoma particularly affects the perifoveal SVP VD (AUROC 0.943, sensitivity 85%, specificity 93%). This could be due to the location of the SVP, which is supplying the RNFL and RGC and therefore the structures most affected by glaucoma^22^.

However, contrary to findings of Takusagawa et al.^17^, which reported that ICP and DCP VD of glaucomatous eyes did not vary significantly from controls, we observed substantial VD reduction in both plexuses (ICP AUROC 0.872, DCP AUROC 0.795). An explanation for this could be the different evaluation techniques applied. While our study relies on an automatic segmentation of the different layers through the manufacturer’s software, they utilized their own segmentation software.

Our results differ from other studies on the assumption that 3 × 3 mm macular scans are seen as disadvantageous for glaucoma diagnosis. Various studies^16,17,23^ argue that this is the case due to the smaller scan window, which could prevent it from imaging the regions most susceptible for glaucomatous vascular changes which typically lie outside of the region captured by 3 × 3 mm scans. While our results demonstrate that perifoveal VD within 3 × 3 mm scans offers diagnostically relevant information, it is worth mentioning that larger scan sizes, such as 6 × 6 mm, could cover additional perifoveal areas beyond those included in smaller scans, potentially offering further information on perifoveal VD. However, our study suggests that it is not mainly about the scan size glaucoma diagnosis is being performed on, but the areas within the scans which are being used for VD analysis, namely the borders and corners of 3 × 3 mm macular scans and therefore the perifoveal region.

Current VD diagnostics of 3 × 3 mm macular OCTA scans is mainly being performed on the parafoveal area^14–16^. However, our results indicate that including perifoveal VD could increase the diagnostic accuracy of glaucoma detection, especially when focusing on the superficial vascular plexus (SVP).

Furthermore, our findings suggest that the diagnostic value of 3 × 3 mm OCTA macular scans may be underestimated. Although larger 6 × 6 mm scans are often perceived as superior to 3 × 3 mm scans in glaucoma detection, especially in detecting early vascular changes^19^, the high diagnostic accuracy of perifoveal VD within this smaller scan window suggests that focusing on the perifoveal area in VD analysis could substantially improve the utility of 3 × 3 mm scans. This would allow clinicians to utilize the faster acquisition speed and higher resolution inherent to 3 × 3 mm scans, which better displays small vessels than larger scans and therefore reveals even subtle vascular changes, while also benefiting from additional diagnostic information of perifoveal VD analysis. This could lead to earlier glaucoma diagnosis and a timelier treatment.

There are multiple limitations to our study. Firstly, we included glaucoma patients but did not differentiate between the different glaucoma types and severity stages available. Future studies should therefore assess the diagnostic accuracy of perifoveal VD compared to parafoveal VD in early, moderate and severe glaucoma and evaluate its use case in different glaucoma types, namely pre-perimetric glaucoma, normal-tension glaucoma, primary open angle glaucoma and glaucoma specific subtypes (e.g. pseudoexfoliation glaucoma).

Secondly, we only included healthy and glaucomatous eyes but did not focus on other eye diseases such as hypertensive or diabetic retinopathy. Since these diseases can also affect the retinal vascular network^24,25^, it would be interesting to see how this would affect the significance of perifoveal VD as a glaucoma-specific biomarker.

Lastly, as current research suggests that 6 × 6 mm scans offer higher diagnostic performance in early glaucoma detection^19^, future work should investigate whether 3 × 3 mm scans are equally capable of identifying early glaucomatous changes when focusing on the perifoveal area in OCTA analysis.

## Summary and conclusion

Building on previous work that highlighted the diagnostic significance of perifoveal areas in deep learning models^13^, our study provides direct evidence that perifoveal VD in 3 × 3 mm OCTA scans offers higher diagnostic accuracy in differentiating healthy from glaucomatous eyes than parafoveal VD, especially when focusing on the SVP.

Our findings suggest that the diagnostic value of 3 × 3 mm scans may be underestimated and proposes to implement the perifoveal region into OCTA VD analysis to further improve glaucoma diagnosis.

Future studies should include other glaucoma types and stages into their evaluation of perifoveal VD and compare whether perifoveal VD in 3 × 3 mm scans can offer equal or higher diagnostic accuracy in detecting early glaucomatous changes than larger scans.

## Data Availability

The data underlying the results is not publicly available due to privacy regulations. Access may be granted by the corresponding author upon reasonable request.
